# Adaptation and psychometric evaluation of the Chinese version of the functional assessment of chronic illness therapy spiritual well-being scale among Chinese childhood cancer patients in China

**DOI:** 10.3389/fpsyg.2022.1065854

**Published:** 2022-12-05

**Authors:** Qi Liu, Ka-Yan Ho, Katherine-Ka-Wai Lam, Winsome Lam, Eileen-Hui-Lin Cheng, Shirley-Siu-Yin Ching, Getaneh Mulualem Belay, Frances-Kam-Yuet Wong

**Affiliations:** School of Nursing, Hong Kong Polytechnic University, Hong Kong, Hong Kong SAR, China

**Keywords:** child, neoplasm, pediatric nursing, spirituality, psychometrics

## Abstract

**Background:**

Spiritual well-being is a strength for childhood cancer patients to cope with cancer. The availability of a valid and reliable instrument for assessing spiritual well-being is crucial. This study translated and adapted the Functional Assessment of Chronic Illness Therapy Spiritual Well-being scale (FACIT-Sp) for Chinese childhood cancer patients and examined the psychometric properties and factor structure in this population.

**Methods:**

This was a methodological study. The FACIT-Sp was translated into Chinese. Adaptation was based on our qualitative study. For psychometric evaluation, a convenience sample of 412 were recruited based on the suggested sample size for the exploratory factor analysis (EFA) and confirmatory factor analysis (CFA). Childhood cancer patients were included if they aged 8–17 years, with parental consent to participate, able to communicate that they were being treated for cancer, and able to communicate and read Chinese. Participants answered the Chinese version of the adapted FACIT-Sp, the Center for Epidemiology Studies Depression Scale for Children (CES-DC), and the Pediatric Quality of Life Inventory 3.0 Cancer Module (PedsQL). Content validity, convergent validity, internal consistency and test–retest reliability were examined. Both EFA and CFA assessed the structural validity of the adapted FACIT-Sp.

**Results:**

The content validity index values for items ranged 0.8–1.0 and that for the scale was 0.84, indicating appropriate content validity. The scale had good internal consistency, with a Cronbach’s alpha of 0.815. The FACIT-Sp scores positively correlated with the CES-DC scores, and negatively correlated with PedsQL scores, suggesting that the Chinese version of the adapted FACIT-Sp had reasonable convergent validity. EFA yielded a four-factor (meaning, peace, faith, and connection with others) model. The CFA results revealed that the four-factor model achieved a better fit than the original three-factor model (Chi-Square Mean/Degree of Freedom = 2.240 vs. 3.557, Comparative Fit Index = 0.953 vs. 0.916, Goodness of Fit Index = 0.909 vs. 0.884, Root Mean Square Error of Approximation = 0.078 vs. 0.112).

**Conclusion:**

The Chinese version of the adapted FACIT-Sp is a reliable and valid instrument for assessing spiritual well-being among Chinese childhood cancer patients. This instrument can be applied in clinical settings for routine assessment.

## Introduction

Cancer is regarded to be a major killer for children worldwide (Steliarova-Foucher et al., 2017). It has been reported that the incidence and mortality rate of childhood cancer in China were 87.1 and 36.3 per million, respectively, after adjusting for age (Zheng et al., 2015). Despite new and advanced treatment options that have improved survival rates, childhood cancer is still regarded as a life-threatening and stressful event (Comas Carbonell et al., 2021). In addition to the physical suffering associated with cancer, the heightened sense of loss and threat of death cause childhood cancer patients to reflect and find meaning in relation to their diagnosis and its impact on their lives (Holge-Hazelton et al., 2016). This ongoing process of reflection and searching throughout the disease trajectory leads to unique spiritual needs among children, which are defined as the needs and expectations of an individual searching for meaning, purpose, and value in their life, and connection with the self, others, the significant, or scared (Murray et al., 2004). Unmet spiritual needs and impaired health status may place childhood cancer patients at increased risk of low spiritual well-being compared with children with other clinical conditions (Taylor et al., 2015).

Spiritual well-being is a fundamental component for the overall quality of life (QoL) of childhood cancer patients (Taylor et al., 2015; Torabi et al., 2018). Spiritual well-being is considered the “fourth domain of well-being,” equally important as physical, mental and social domains (Dhar et al., 2013). It refers to a state of being where an individual is able to handle daily life issues in a way that leads to personal realization of one’s full potential, meaning and purpose of life and fulfilment from within (Aging, 1975). Spiritual well-being is regarded as a source of strength for childhood cancer patients by providing meaning and purpose, and fostering strong relationships with others and with the divine, hence heightening patients’ resistance to the suffering caused by disease (Hendricks-Ferguson, 2008; Foster et al., 2012). Mounting evidence has shown that high spiritual well-being can act as a buffer against depressive symptoms and anxiety in patients with cancer (Chaar et al., 2018; Davis et al., 2018), including those who are adolescent and young adults (Grossoehme et al., 2020). Therefore, healthcare professionals should develop and implement appropriate interventions to improve spiritual well-being among childhood cancer patients. However, before any intervention can be properly designed and evaluated, it is essential to have access to a valid and reliable instrument for assessing spiritual well-being among pediatric cancer patients.

A previous systematic review evaluated the measurements of spirituality in adolescent health outcomes research, and identified five most commonly used scales to assess spiritual well-being, including Brief Multidimensional Measure of Religiousness/Spirituality, Spiritual Well-Being Scale, Religious Coping Questionnaire, Religious Orientation Scale, and Systems of Belief Inventory (Cotton et al., 2010). However, two limitations are needed to be considered when selecting a spiritual scale for childhood cancer patients. Firstly, these scales contained more than half of items on religion, which may not be suitable for non-religious children. Although religion usually links with the spirituality, spiritual well-being can be achieved for both religious population and non-religious population. Patients with religion may attain spiritual well-being *via* praying to god and finding connections with a supreme being, thus gaining comfort and enhancing their willingness to actively seek treatment (Peteet and Balboni, 2013). While for non-religious patients, like those in China, often rely on other means, such as self-exploration and connections with their loved ones, to identify the meaning of their existence and suffering, giving them the strength to continue their struggle against cancer (Peteet and Balboni, 2013). Hence, it is necessary to identify and select a scale that is appropriate to assess spiritual well-being for both religious and non-religious childhood cancer patients. Secondly, most studies using these five scales did not report the relevant psychometrics in adolescent population (Cotton et al., 2010). Importantly, none of these five scales have been rigorously validated in childhood cancer population.

The Functional Assessment of Chronic Illness Therapy Spiritual Well-being Scale (FACIT-Sp) is the most often employed measure for assessing spiritual well-being in adult cancer patients (Monod et al., 2011). The FACIT-Sp was developed based on interviews with American cancer patients and oncology specialists, and further validated in patients with different religions and in non-religious patients in various contexts, including Brazil, Denmark, Korea, Iran, Jordan, Norway and the United States (Bredle et al., 2011). The scale contains 12 items, and confirmatory factor analysis (CFA) revealed three scale domains: (1) the meaning domain, which assesses meaning and purpose in life; (2) the peace domain, which assesses the feeling of peace and calmness; and (3) the faith domain, which assesses the faith derived from religion or other sources (e.g., supernatural power) (Canada et al., 2008). The FACIT-Sp has been utilized to evaluate spiritual well-being among pediatric patients (Cotton et al., 2009, 2012). Evidence indicates that this instrument is reliable and valid for use with adolescents with chronic illness (de Alvarenga et al., 2019). However, the FACIT-Sp has never been validated for use with childhood cancer patients, including in the Chinese context. Because of the uncertain prognosis, long-term hospitalization, and difficult treatment, childhood cancer patients have unique spiritual needs, which distinct from those with other diseases, according to previous studies (Holge-Hazelton et al., 2016). Importantly, culture plays a significant role in influencing spiritual needs (Conner and Eller, 2004). Due to the aforementioned concerns, it may be premature to directly apply the FACIT-Sp to evaluate the spiritual well-being of childhood cancer patients prior to adaptation and psychometric testing. There is currently no validated instrument for assessing spiritual well-being in children with cancer. As such, the current study aimed to translate and adapt the FACIT-Sp for Chinese children with cancer and examine the psychometric properties and factor structure in this population.

## Materials and methods

### Study design

This study was a methodological study that was done cross-sectionally. This study was conducted in three stages: translation of the FACIT-SP scale, adaption of the FACIT-Sp scale based on our qualitative results of spiritual needs among Chinese childhood cancer patients, and psychometric evaluation of the adapted scale.

### Translation

For the purpose of translation and adaption, an expert panel was established. The translation process followed the method proposed by Bracken and Barona (1991). Initially, the English version of the FACIT-Sp was translated into the Chinese version by two bilingual translators independently. The panel member then compared and reconciled the two translations. The back-translation was completed by two additional independent bilingual translators who were blinded to the original English version of the scale. The panel members checked to see if the original meaning of each item of the scale was preserved in the back translations by comparing them to the original English version. Disagreement were settled at regularly scheduled meetings.

### Adaption

The adaptation of the FACIT-Sp was based on a qualitative study which explored the spiritual needs and concerns in Chinese childhood cancer patients aged 8–17 years from a pediatric public hospital. A descriptive phenomenological qualitative was applied. A total of 22 childhood cancer patients recruited by purposive sampling were invited to join an individual semi-structured interview. The semi-structured interview guide covered three aspects of spirituality, including meaning and purpose, relationship, and religion and faith. Example of questions included “Which experience in your cancer journey makes you feel fulfilled?” and “What is the impact of spirituality on your cancer journey?.” The data was analyzed using the thematic analysis. Details of this qualitative study has been previously published in elsewhere (Liu et al., 2022). In brief, patients’ spiritual needs can be divided into four categories: (1) meaning of cancer in their life, (2) personal inner needs, (3) connections with others, and (4) connections with gods, supernatural powers and fictional characters.

Based on the findings of our qualitative research, we found that three identified categories (meaning of cancer in their life, personal inner needs, and connections with gods, supernatural powers and fictional characters) corresponded to the three domains of the FACIT-Sp (meaning, peace, and faith domains). However, the remaining category (connections with others) in our qualitative study was not adequately assessed in the original FACIT-Sp. Thus, in accord with previous literature and our qualitative findings, we added three items to assess the new “connections with others” category: (1) I feel free to express my concerns to others; (2) I can feel the support of others; and (3) I think I am still helpful to others.

Previous evidence suggested that at least five participants are needed to test the comprehension and identify possible ambiguous aspects of scales for children (Halliwell et al., 2017; Llistosella et al., 2019; Riad et al., 2021). Hence, the preliminary version was administered to 10 hospitalized cancer patients (selected based on the same inclusion criteria for the participants in the psychometric evaluation) to test the comprehension of the adapted scale for children.

## Psychometric evaluation

### Setting

The study was conducted in Hunan Children’s Hospital in South-central China, which provides treatment to all children with cancer in the province. The selection of this study site ensured the representativeness of our participants. The Institutional Review Board of Hong Kong Polytechnic University approved this research (HSEARS20220127001).

### Participants

Hospitalized childhood cancer patients were recruited through convenience sampling. The eligibility criteria were as follows: (1) age: 8–17 years old; (2) with parental consent to participate, (3) diagnosed with any cancer and were undergoing treatment at any stage (Grossoehme et al., 2020); (4) ability to communicate that they were having cancer treatment; and (4) ability to communicate and read Chinese. Individuals with identified cognitive and/or behavioral problems that affected communication were excluded. This age range (8–17 years old) was chosen based on the childhood cognitive development. According to the Piaget theory of childhood cognitive developmental stages (Ramesh, 2022), children aged 0–7 years have not developed the mental capacities of logic thought. These immature mental capacities may prevent them from generating an explanation of their own experience, which could impede their understanding of questions in the scale. Thus, only those ≥8 years old were selected because they could comprehend the questionnaire.

Although there are no clear guidelines regarding sample size calculation for factor analysis, ≥200 participants are required for CFA (Gorsuch, 2014). Also ≥200 participants are needed for EFA (Sakaluk and Short, 2017). We therefore determined to recruit 400 hospitalized childhood cancer patients in the current study.

### Study instruments

#### Socio-demographic and clinical characteristics

Participants’ demographic and clinical variables, including age, sex, parental educational attainment, household size, religious affiliation, diagnosis, and treatment type, were documented on an information sheet.

#### The Chinese version of the adapted FACIT-Sp scale

The adapted FACIT-Sp assessed individuals’ spiritual well-being in the last 7 days. It contains 15 items that can be further categorized into four domains: the meaning domain, peace domain, faith domain, and connections with others domain. All items are scored between 0 (“not at all”) and 4 (“very much”) on a five-point rating scale. The scores are added together to get a total score that ranges from 0 to 60, with higher scores indicating greater spiritual well-being. Previous psychometric testing of the English version of the FACIT-Sp have verified its reliability and validity for measuring spiritual well-being among adult cancer patients (Canada et al., 2008).

#### The Chinese version of the center for epidemiology studies depression scale for children

The CES-DC was used to record the depressive symptoms experienced by the participants. This questionnaire has 20 items, each of which is rated on a four-point Likert scale based on the last week’s experiences. The range of scores is from 0 to 60, with higher scores indicating more depressive symptoms. The cut-off score is 16, suggesting that children exhibit a considerable number of depressed symptoms. The psychometric properties of the Chinese version of the CES-DC have been reported to show good internal consistency (the Cronbach’s α = 0.82), and appropriate convergent and discriminant validity (Li et al., 2010).

#### The Chinese version of the pediatric quality of life inventory 3.0 cancer module

Using the PedsQL, the quality of life of the participants was measured. There are a total of 27 questions on this scale, which are divided into eight subscales: pain and hurt, nausea, procedural anxiety, treatment anxiety, worry, cognitive problems, perceived physical appearance, and communication. The PedsQL scores vary from 0 to 100, with higher values indicating a higher quality of life in terms of one’s health. The scale shows good reliability with a Cronbach’s α value of 0.87 and validity in Chinese Children with cancer (Ji et al., 2011).

### Data collection

First, a research assistant approached the parents of eligible patients in pediatric wards, and explained the design, method, and potential implications in detail. Parents who consented to their children’s participation were requested to sign a written consent. Both the parents and the children were given the assurance that they could stop participating at any point without penalty. All participants were required to fill in the adapted FACIT-Sp, CES-DC, and PedsQL 3.0 Cancer Module. The Peds-QL and CES-DC were included for examining the convergent validity, since spiritual well-being relates to both quality of life and depressive symptoms (Bai et al., 2015; Chaar et al., 2018). For the purpose of evaluating test–retest reliability, a total of 50 hospitalized pediatric cancer patients were invited (with parental consent) to complete the adapted FACIT-Sp again after 2 weeks (Hopkins, 2000; Li et al., 2010; Carvajal et al., 2011; Ho et al., 2015, 2021).

### Data analysis

#### Semantic equivalence

Semantic equivalence of the translated items was evaluated. A panel of experts was asked to score the translation equivalence between the original English and Chinese versions of the FACIT-SP for each item on a four-point scale (from 1 = not equivalent to 4 = most equivalent). The equivalence rate (the proportion of the total items rated by the experts as either 3 or 4) would next be computed. Any item that received a rating of 1 or 2 from more than 20% of the panel members would be revised.

#### Content validity

The content validity of the Chinese version of the adapted FACIT-Sp was evaluated using the content validity index (CVI). According to the item relevance to the concept of spiritual well-being, the expert panel graded each item from 1 (entirely irrelevant) to 4 (highly relevant). For each item, the CVI of an item (I-CVI) was determined by dividing the proportion of experts who gave a rating of 3 or 4 of experts who rated the item. Good content validity was indicated by I-CVI ≥ 0.80 (Polit and Beck, 2006). Items rated below 0.8 were discussed and considered for deletion or revision.

#### Reliability testing

Cronbach’s alpha was used to evaluate the internal consistency reliability of the Chinese versions of the adapted FACIT-Sp, and values over 0.70 were regarded to be satisfactory. The intra-class correlation (ICC) analysis was used and the ICC coefficient was calculated to examine test–retest reliability. ICC values lower than 0.40 were considered weak, values of 0.41–0.59 were considered moderate, values of 0.60–0.75 were considered good or substantial, and values higher than 0.75 were considered very good (Mcdowell, 2006).

#### Convergent validity

Convergent validity was established by finding correlations between scores on the Chinese versions of the adapted FACIT-Sp and CES-DC, as well as the PedsQL 3.0 Cancer Module using Spearman’s correlation coefficients. On the basis of previous literature (Bai et al., 2015; Chaar et al., 2018), we hypothesized that adapted FACIT-Sp scores would have a positive correlation with PedsQL 3.0 scores and a negative correlation with CES-DC scores.

#### Exploratory factor analysis and confirmatory factor analysis

To examine the underlying factor structure of the Chinese version of the adapted FACIT-Sp, EFA was first conducted, followed by CFA. These analyses were used to ascertain that the factor structure found in the EFA adequately fit the data. Because the EFA and CFA were needed to be performed on two different datasets, the original dataset (*N* = 412) was split in half randomly (datasets A and B). EFA and CFA were performed on datasets A (*N* = 206) and B (*N* = 206), respectively.

EFA was carried out utilizing the Statistical Package for Social Sciences (SPSS) software, version 26.0 for Windows (SPSS Inc., Chicago, IL, United States). Prior to EFA, data sufficiency was confirmed using Bartlett’s test of sphericity and the Kaiser–Meyer–Olkin test. A principal components analysis was then performed. Coefficients equal or greater than 1.0 according to Kaiser’s criterion were considered (Kline, 2014). For the screen test, all factors above the elbow, or the break in the plot, were kept, since they accounted for a larger portion of the total variance in the data set (Cattell, 1966).

After the EFA analysis, CFA was performed using AMOS for Windows, version 20.0. The overall fit of the three-factor (meaning, peace and faith) and four-factor (meaning, peace, faith, and connection with others) models were examined and compared. The overall fit of the models were examined using goodness of fit indices, including the χ2/degrees of freedom (df) ratio, goodness of fit index (GFI), normed fit index (NFI), adjusted goodness of fit index (AGFI), comparative fit index (CFI), incremental fit index (IFI), and root mean square error of approximation (RMSEA) (Kline, 2014).

## Results

### Demographic and clinical characteristics

Table 1 presents participants’ demographic and clinical characteristics. Participants’ mean age was 12.3 years (standard deviation [SD] = 2.9) and the mean household size was 4.14 (SD = 1.1). Of the participants, 56.3% (*n* = 232) were girls, 86.8% (*n* = 357) were non-religious, 52.2% (*n* = 215) of parents had attained upper secondary school education, 66.8% (*n* = 275) were diagnosed with non-solid tumor, 52.9% (*n* = 232) were diagnosed within 6 months, and 20.8% (*n* = 86) had received multiple treatments for cancer.

**Table 1:** Sociodemographic and clinical characteristics of the participants (*N* = 412).

	*n* (%)
**Age range**	
8–12 years	210 (51.0)
13–18 years	202 (49.0)
**Gender**	
Male	180 (43.7)
Female	232 (56.3)
**Parents’ educational attainment**	
Lower secondary school or below	197 (47.8)
Upper secondary school or above	215 (52.2)
**Household size**	
1–3	149 (36.2)
4–5	226 (54.9)
>5	37 (9.0)
**Diagnosis**	
Non-solid tumor	275 (66.8)
Solid tumor	137 (33.2)
**Time since diagnosis**	
< 6 months	218 (52.9)
6–12 months	102 (24.8)
>1 year	92 (22.3)
**Treatment received**	
Surgery	41 (10)
Chemotherapy	259 (62.9)
Bone marrow transplant	26 (6.3)
Mixed method	86 (20.8)
**Home religious’ affiliation**	
With religion	55 (13.2)
No religion	357 (86.8)

### Semantic equivalence

For the translated items, the semantic equivalence ranged from 0.8 to 1.0. The overall rate was 0.83, indicating that each item of the Chinese version of the FACIT-Sp was equivalent to those in the English version in both conceptual and idiomatic terms.

### Content validity

For the Chinese version of the adapted FACIT-Sp scale, the I-CVIs values ranged from 0.8 to 1, and the S-CVI was 0.84 for the overall scale, indicating that the adapted scale had satisfactory content validity.

### Reliability

The internal consistency of the adapted scale was measured using a Cronbach’s alpha of 0.815. The corrected item–total correlations ranged from 0.29 to 0.70, indicating acceptable internal consistency. At the 2-week interval, the test–retest reliability coefficient was 0.79 (*p* < 0.001).

### Convergent validity

Spearman’s correlation coefficients of 0.64 and 0.47 were found between the scores of FACIT-Sp and CES-DC, and between the scores of FACIT-Sp and PedsQL, respectively. The results indicated that the translated and adapted version of the FACIT-Sp had appropriate construct validity.

### Exploratory factor analysis

The Kaiser–Meyer–Olkin value was 0.807, and the results of Bartlett’s test of sphericity were significant (p < 0.001), revealing that the dataset met was suitable for factor analysis. Four factors with an eigenvalue higher than 1 were identified using principal components analysis, which explained 72.72% of the total variance. Factor 1 (“meaning”) explained 17.66% of the variance, factor 2 (“peace”) explained 22.25% of the variance, factor 3 (“faith”) explained 17.52% of the variance, and factor 4 (“connection with others”) explained 15.29% of the variance. All items were loaded on a single dominating factor and had factor loadings greater than 0.4. The interpretation of three components (meaning, peace, and faith) was consistent with the proposed factor structures of the original English version of the FACIT-Sp. Three newly added items successfully loaded on the “connection with others” factor (Table 2).

### Confirmatory factor analysis

**Figure 1:**
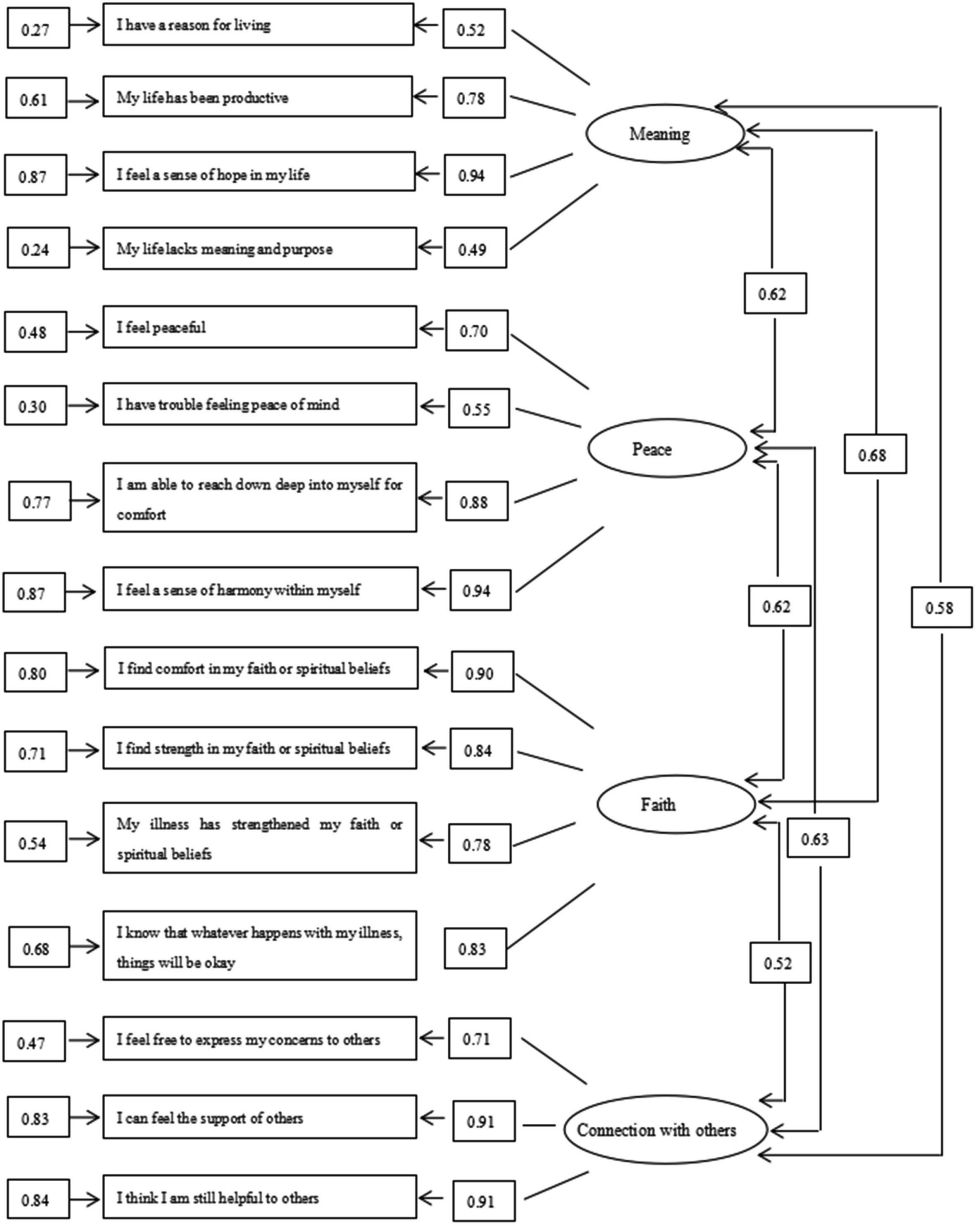
Confirmatory factor analysis.

The parameter estimates of the four-factor model are displayed in Figure 1. All correlation matrices were positively definite and were less than 1, indicating that the parameter estimates were appropriate. The observed variables all had high factor loadings, ranging from 0.49 to 0.94. The t-values of all variables were higher than 1.96, indicating that the loadings on the variables were significant.

The overall fit of the three-factor model based on 12 items of the original FACIT-Sp, and the four-factor model based on 15 items of the adapted version were tested by a variety of fit indices. The results suggested that the four-factor model based on 15 items was better than the three-factor model based on 12 items (Tables 3, 4).

**Table 2:** Exploratory factor analysis of the 15-item FACIT-Sp scale (*n* = 206).

Items	Component 1 meaning	Component 2 peace	Component 3 faith	Component 4 connection with others
I have a reason for living	0.759			
My life has been productive	0.744			
I feel a sense of hope in my life	0.653			
My life lacks meaning and purpose	0.820			
I feel peaceful		0.703		
I have trouble feeling peace of mind		0.755		
I am able to reach down deep into myself for comfort		0.765		
I feel a sense of harmony within myself		0.723		
I find comfort in my faith or spiritual beliefs			0.817	
I find strength in my faith or spiritual beliefs			0.764	
My illness has strengthened my faith or spiritual beliefs			0.852	
I know that whatever happens with my illness, things will be okay			0.708	
I feel free to express my concerns to others				0.847
I can feel the support of others				0.830
I think I am still helpful to others				0.830

**Table 3:** Fit statistics for the 12-item 3-factor and 15-item 4-factor model.

Factor model	*χ2* /df	CFI	GFI	RMSEA
15-item 4-factor model	2.240	0.953	0.909	0.078
12-item 3-factor model	3.557	0.916	0.884	0.112

χ2/df, Chi-square mean/degree of freedom; RMSEA, root mean square error of approximation; CFI, comparative fit index; FI, goodness of fit index.

**Table 4:** Acceptable overall fit of each model was evaluated using the following indices.

Criteria	Range
χ2 /df	3.00–5.00
CFI	0.9 or higher
GFI	0.9 or higher
RMSEA	0.08 or less

## Discussion

Spiritual well-being was recently recognized as the fourth dimension of health by the World Health Organization (Dhar et al., 2013), and it is reported to be closely linked to physical and psychosocial well-being in patients with childhood cancer (Grossoehme et al., 2020). Nevertheless, there is currently no validated instrument for assessing spiritual well-being in children with cancer. To address this gap in existing literature, we translated and adapted the FACIT-Sp, a widely used instrument for assessing spiritual well-being in adult cancer patients. Additionally, the psychometric properties of the adapted Chinese version were tested in Chinese children who were hospitalized with cancer in China.

In accord with previous validation studies of adolescents with chronic diseases and young adults with cancer (de Alvarenga et al., 2019; Grossoehme et al., 2020), the Chinese version of the adapted FACIT-Sp showed satisfactory internal consistency. Specifically, all items of the adapted FACIT-Sp appeared to measure the same construct, spiritual well-being, with the corrected item-total correlations ranging from 0.29 to 0.70. In addition, the ICC at 2 weeks was high, indicating good test–retest reliability.

Regarding content validity, most items in the adapted Chinese version of the FACIT-Sp were found to be reflective of the underlying construct (spiritual well-being). However, when we piloted the prototype scale in Chinese children with cancer, few respondents reported (*n* = 2) that item 5 (“I feel a sense of purpose in life”) was difficult to answer. These two respondents felt that the purpose of life was an ambiguous concept for them to consider, particularly because they were very young to be thinking about this issue. Hence, even if participants responded “not at all,” this did not necessarily indicate that they thought their life was purposeless. Interestingly, these two respondents also stated that they were on a path of searching for their purpose in life, and that the journey of combatting cancer provided them with an opportunity to think and identify their life purpose. Thus, cancer appeared to serve as a catalyst for some children to find purpose, although they still felt unsure of their specific purpose in life. Given the importance of life purpose in the concept of spiritual well-being (Lormans et al., 2021), the panel members decided, after thorough discussion, to retain this item for validation. This item also showed satisfactory factor loadings in both EFA and CFA. However, future research will be required to determine whether refinement of the item wording is necessary to improve clarity for children.

A negative correlation was found between the adapted FACIT-Sp and the CES-DC scores, providing evidence for the convergent validity. These findings were in accord with previous studies reporting that childhood cancer patients with higher levels of spiritual well-being presented fewer depressive symptoms and had better QoL (Bai et al., 2015; Chaar et al., 2018).

The EFA findings indicated that the four-factor model explained 72.72% of the total variance, which was over the Streiner-suggested threshold of 50% (Streiner, 1994). In addition, the CFA results revealed that the four-factor model provided a better fit than the original three-factor model. This result suggests that the four-factor model with the inclusion of “connection to others” domain better captures the concept of spiritual well-being of Chinese childhood cancer patients than the original three-factor model. Unlike adult cancer patients who require room for self-reflection on life goals and values (Van Gurp et al., 2020), children with cancer are more likely to rely on family and friends to establish normalcy in their disease trajectory (Hay and Nye, 2006). The intimate relationships between children and their families as well as their friends serve as a source of love, compassion, distraction, and support to motivate them to combat the disease (Petersen, 2014). Furthermore, in contrast to children in Western countries who relieve their spiritual distress by finding a connection with God *via* engaging in religious practices, Chinese children count more on the connection with people surrounding them (Moore et al., 2020). This could be because most Chinese are not religious, with only 15% of Chinese having religious affiliations (Zhang and Lu, 2020). The results from our previous qualitative study indicated that there were three ways adopted by Chinese childhood cancer patients to establish the connection with others. The three ways included expressing their suffering and emotional distress to their loved ones, feeling of being supported by others, and being helpful to others (Liu et al., 2022). Expressing suffering to their loved ones help them to share and manage their losses, concerns and feelings, thus alleviating their spiritual suffering (Petersen, 2014; Liu et al., 2022). Feeling of being supported by others can serve as a spiritual support to motivate them to continue the treatment, notwithstanding the suffering caused by cancer (Petersen, 2014; Liu et al., 2022). Being helpful to others allows them to gain appreciations from others, so that they can find their self-value even in this difficult journey (Liu et al., 2022). These three ways of “connection with others” can assist them to overcome an existential crisis, ultimately improving their spiritual well-being. Hence, adding the new domain “connection with others” with its three items in the FACIT-Sp can greatly enhance its structural validity to capture spiritual well-being of children with cancer in the Chinese context.

Most items had significant factor loadings in the four-factor model, as indicated by the CFA results, with the exception of item 4 (“My life lacks meaning and purpose”) that attained a factor loading of 0.49. This is accords with the results of previous validation studies of the FACIT-Sp in adult populations (Canada et al., 2008; Murphy et al., 2010; Lazenby et al., 2013). The low factor loading for item 4 may have been caused by this item being negatively worded. Increasing evidence suggests that negatively worded items do not match the overall logic of other items that are positively worded, and hence people may respond incorrectly if they do not pay sufficient attention to distinguishing negatively worded items from positively worded items (Sonderen et al., 2013; Salazar, 2015). Indeed, “careless responses” and “difficulty in understanding” have been persistently acknowledged as the major reasons for low factor loadings for negatively worded items in previous studies (Salazar, 2015). Although some researchers have suggested switching out negatively worded items on the FACIT-Sp for positively worded ones (Murphy et al., 2010; Lazenby et al., 2013), caution must be taken because negatively worded items can reduce response set bias, which is defined as the tendency of respondents to answer similarly to all or many items (Van Herk et al., 2004). The think-aloud approach suggested by Lewis could be adopted in further studies to give recommendations regarding if the negatively phrased items in the FACIT-Sp should be retained or replaced (Güss, 2018). In particular, respondents can be asked to verbalize or write down their thoughts when answering each item (Güss, 2018). Researchers can then make use of these think-aloud data to specify questions that are easily misinterpreted and even develop further questions for clarification, thus obtaining more accurate responses (Güss, 2018).

### Implications for future practice

Increasing evidence indicates that low levels of spiritual well-being are strongly related to the development of various psychological symptoms, such as depression and anxiety, in children with different clinical conditions including cancer (Taylor et al., 2015; Pratiwi et al., 2018). Given the importance of spiritual well-being in the disease trajectory, the Chinese version of the adapted FACIT-Sp can be used as a standard evaluation instrument, and early intervention can be provided to minimize its associated healthcare costs. Additionally, previous studies have developed interventions that can improve spiritual well-being for adult cancer patients (Kruizinga et al., 2016). However, due to the lack of a reliable instrument to evaluate spiritual well-being for children with cancer, including those in China, the development of spiritual interventions is lagging far behind. Hence, to advance clinical practice, the adapted FACIT-Sp may be useful for evaluating the effectiveness of existing spiritual interventions, improving the spiritual well-being of Chinese children hospitalized with cancer.

### Limitations

There are several limitations should be noted in the current study. First, the generalizability of the results may restrict by the using of convenience sampling. Second, because of ethical considerations, childhood cancer patients who were terminally ill were not recruited in this study. It is therefore unclear whether this adapted scale can be used to evaluate the spiritual well-being of Chinese children with terminal cancer.

## Conclusion

The current study addressed a gap in the literature by translating and adapting the FACIT-Sp for Chinese children with cancer. Statistical analyses confirmed that the adapted FACIT-Sp is a reliable and valid instrument for assessing spiritual well-being in this population.

## Data availability statement

The raw data supporting the conclusions of this article will be made available by the authors, without undue reservation.

## Ethics statement

The studies involving human participants were reviewed and approved by the Institutional Review Board, Hong Kong Polytechnic University. Written informed consent to participate in this study was provided by the participants’ legal guardian/next of kin.

## Author contributions

QL: conceptualization, methodology, data collection, and draft preparation. K-YH: conceptualization, methodology, draft preparation, and supervision. GB: revising and editing. K-K-WL: conceptualization, methodology, and writing. E-H-LC: writing—review and editing. S-S-YC: conceptualization, writing—review and editing. F-K-YW: writing—review and editing. WL: writing—review and editing. All authors contributed to the article and approved the submitted version.

## Conflict of interest

The authors declare that the research was conducted in the absence of any commercial or financial relationships that could be construed as a potential conflict of interest.

## Publisher’s note

All claims expressed in this article are solely those of the authors and do not necessarily represent those of their affiliated organizations, or those of the publisher, the editors and the reviewers. Any product that may be evaluated in this article, or claim that may be made by its manufacturer, is not guaranteed or endorsed by the publisher.
